# Understanding African swine fever dynamics in South Korean pig farms through agent-based modeling and spatiotemporal analysis of farm outbreaks

**DOI:** 10.3389/fvets.2026.1945299

**Published:** 2026-10-22

**Authors:** Maryum Tanveer, Fabrice Hirwa, Eurade Ntakiyisumba, Gayeon Won

**Affiliations:** College of Veterinary Medicine and Bio-Safety Research Institute, Jeonbuk National University, Iksan, Republic of Korea

**Keywords:** African swine fever (ASF), agent-based modeling, disease spread dynamics, outbreak patterns, spatiotemporal analysis

## Abstract

African swine fever continues to pose a major threat to the swine industry in South Korea since 2019, with increased farm outbreaks in 2026. This study applied agent-based modeling and spatiotemporal outbreak analysis to characterize ASF dynamics at within-farm and between-farm levels. A stochastic SEIR agent-based model was developed to simulate within-farm transmission through direct pig-to-pig contact and indirect environmental pathways under varying epidemiological and biosecurity conditions. For the between-farm component, spatiotemporal patterns of confirmed farm outbreaks from 2019 to 2026 were analyzed, with particular emphasis on the 2026 epidemic. Notably, the 2026 outbreak pattern showed early multifocal occurrence, followed by regional clustering and broader geographic dispersion. Distance-based analysis of temporally ordered confirmed outbreaks showed a median nearest-neighbor distance of approximately 31 km, with several outbreaks located >100 km from the nearest previously confirmed outbreak, including during the early phase of the epidemic. These findings are compatible with a multi-pathway interpretation in which local clustering, delayed detection, unobserved intermediate infections, and non-local production-network-associated pathways may all contribute to observed outbreak patterns. This combined analytical approach provides a more cautious and comprehensive understanding of ASF dynamics in South Korean pig farms and highlights the need for control strategies that couple strong farm-level biosecurity and rapid local response with broader surveillance and risk management of high-connectivity points in the pig production system.

## 1 Introduction

African swine fever (ASF) is a highly contagious and severe hemorrhagic disease of domestic pigs and wild boars, characterized by high morbidity and case-fatality rates that can reach nearly 100% in acute forms, posing a profound threat to global swine production (1, 2). Since its initial identification in Kenya in the 1920s, ASF has re-emerged periodically in sub-Saharan Africa and has repeatedly spread beyond the continent, causing outbreaks in Europe and South America. Although the disease was largely eradicated from these regions, Sardinia, Italy, remained a notable exception with long-term endemicity. A major epidemiological shift occurred following its introduction into Georgia in 2007, after which the virus became widely established among domestic pigs and wild boar across Eastern Europe and parts of the Russian Federation (3, 4).

Due to its large double-stranded DNA genome and protective lipid envelope, the ASF virus (ASFV) exhibits remarkable environmental resilience and can persist in pork products, fomites, and contaminated materials. This persistence facilitates transmission through direct contact and indirect environmental exposure, thereby complicating disease control measures and contributing to the potential for spread through trade- and movement-related pathways (5, 6). The lack of effective preventive tools, such as commercially viable vaccines or antiviral drugs for ASF, further underscores the importance of stringent biosecurity measures and early detection strategies to reduce disease transmission and support effective outbreak management (7, 8).

In South Korea, pig farming constitutes a vital component of the agricultural sector, accounting for approximately 30% of livestock production value, with annual pork production exceeding 1 million metric tons (9–11). Consequently, the occurrence of ASF in domestic pig farms presents substantial challenges that threaten animal health and have profound economic consequences for the swine industry (12, 13). This concern originated in September 2019, when South Korea confirmed its first domestic pig outbreak in Gyeonggi-do province (14). Since then, 79 outbreaks on domestic farms have been reported in South Korea as of March 2026 (15, 16). In the first three months of 2026, 24 outbreaks were reported in domestic pig farms across multiple provinces of South Korea, including Gyeonggi-do (7), Gyeongsangnam-do (5), Jeollanam-do (4), Chungcheongnam-do (3), Jeollabuk-do (2), Gangwon-do (2), and Gyeongsangbuk-do (1). Notably, several outbreaks were confirmed in areas geographically distant from earlier confirmed outbreaks, indicating a geographically dispersed outbreak pattern during the early phase of the 2026 epidemic (17).

As of April 2025, there are 5,385 registered pig farms in South Korea, collectively housing an estimated 10.8 million domestic pigs (9–11). However, the number of domestic pigs raised per farm varies substantially by scale; small-scale farms typically house fewer than 1,000 pigs, medium-scale farms manage between 1,000 and 5,000 pigs, and large-scale or industrial farms can hold 5,000 ~ 10,000 pigs or more (18–20). High farm density, interconnected supply chains, indirect contact through contaminated fomites, and the wild boar–domestic pig interface may collectively increase the risk of ASF occurrence and spread in the region (21, 22). The continued occurrence of ASF outbreaks in South Korea despite the implementation of culling and surveillance strategies suggests potential limitations in current control measures, including the absence of an effective vaccine, insufficient biosecurity practices, constraints in the effectiveness of culling-based interventions, and delays in early detection and reporting mechanisms (23, 24). A detailed understanding of both within-farm transmission processes and between-farm outbreak patterns is thus essential for informing more effective, evidence-based control strategies (25).

While compartmental models such as SEIR are useful for describing population-level trends of ASF, they are limited in their ability to represent the heterogeneity and stochasticity inherent in disease transmission within complex pig farming systems (26). This limitation is particularly relevant in South Korea, where farm structures, biosecurity practices, and movement patterns vary substantially. Existing studies have described the spatial proliferation of ASF in wild boar and reported farm-level outbreaks, but quantitative studies that consider both within-farm transmission dynamics and between-farm outbreak patterns in the Korean domestic pig sector remain limited. To address these gaps, the present study applies an agent-based modeling (ABM) framework for within-farm transmission alongside spatiotemporal and distance-based analyses of confirmed farm-level outbreaks. The stochastic susceptible–exposed–infectious–removed agent-based model explicitly represents individual pigs and simulates infection via direct contact and indirect environmental pathways under a range of epidemiological and biosecurity conditions. For the between-farm component, confirmed ASF outbreaks in domestic pig farms from 2019 to 2026, with particular emphasis on the 2026 epidemic, were analyzed to characterize temporal patterns, spatial distribution, and distance-based outbreak patterns among temporally ordered confirmed outbreaks (27, 28). This two-component analytical approach provides complementary insights into simulated within-farm epidemic behavior and observed farm-level outbreak patterns, while avoiding assumptions about confirmed farm-to-farm transmission links (29, 30). Accordingly, the study aims to quantify how within-farm transmission parameters and control measures influence outbreak size and duration, describe the spatiotemporal progression and distance-based patterns of recent confirmed ASF outbreaks in domestic pig farms in South Korea, and discuss control implications that combine farm-level biosecurity, rapid local response, and broader surveillance of high-connectivity points within the pig production system.

## 2 Research method

### 2.1 Within-farm transmission model

#### 2.1.1 Model overview and design

To simulate the transmission dynamics of ASF among pigs within a single farm, a stochastic ABM was developed based on a susceptible–exposed–infected–removed (SEIR) disease structure. The model was implemented in NetLogo (*version 6.4*), an agent-based modeling environment designed for simulating the behavior and interactions of individual agents within complex systems (31, 32). The model structure was adapted from the NetLogo epiDEM Basic model, a previously developed epidemiological simulation framework for exploring infectious disease transmission dynamics (33). The original framework was extended by incorporating an explicit latent (E) stage and a hazard-based force of infection that includes both direct pig-to-pig transmission and indirect transmission through environmental contamination, thereby capturing key within-farm ASF dynamics (Figure 1). Simulations were conducted using a daily time step (1 tick = 1 day), consistent with the biological timescale of ASF infection and progression.

**Figure 1:**
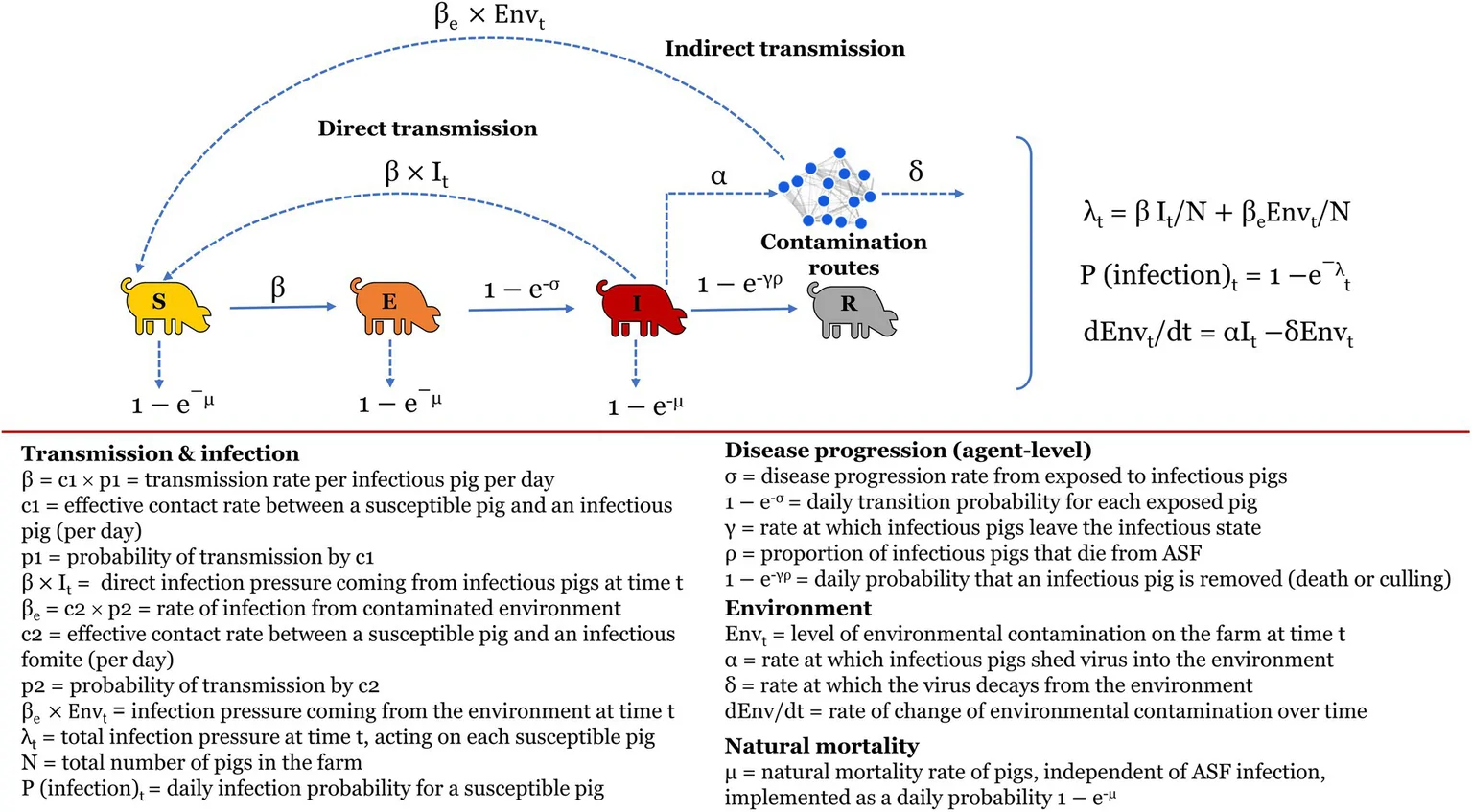
Schematic representation of the within-farm agent-based model for African swine fever transmission, incorporating direct pig-to-pig transmission and indirect environment-mediated transmission.

Model stochasticity was introduced through probabilistic infection events and state transitions at the individual level, with parameters sampled from predefined ranges (Table 1). Simulation experiments were performed using the BehaviorSpace, an integrated NetLogo tool that enables repeated model runs while systematically varying parameter values and recording model outputs (32). To account for parameter uncertainty, values were sampled using Latin hypercube sampling (LHS) based on published epidemiological estimates or plausible assumptions. A total of 1,000 simulations were conducted, each representing a unique parameter combination, enabling systematic evaluation of the influence of parameter uncertainty on model outcomes (Supplementary Tables S1–S3). Model validation was conducted by comparing the simulated outputs (e.g., R0, epidemic duration, cumulative mortality, and peak prevalence) with published epidemiological data from ASF outbreaks in South Korea and other regions.

**Table 1:** Key epidemiological parameters used for modeling within-farm transmission of African swine fever in South Korean pig production systems.

Parameter name	Symbol	Value/Formula	Source
Within-farm transmission model (Baseline scenario)			
Farm size	N	500–2,500	Range informed by South Korean pig farm size statistics (10)
ASF Incubation period (days)	IP	4–19	(67–69)
ASF Latent period (days)	LP	2–6	(70, 71)
Duration of the Infectious period (days)	D	3–10	(72, 73)
Pig-to-pig transmission rate (per day)	β	c_1_ × p_1_	Calculated using the formula described in (72, 74)
Effective daily contacts between susceptible and infectious pigs	c_1_	Poisson (1)	Assumed contact frequency informed by published ASF transmission evidence (75)
Probability of transmission by c_1_	p_1_	0.05–0.25	Range selected based on published estimates of ASFV transmission probability (72, 74, 76)
Environment-to-pig transmission rate (per day)	β_e_	c_2_ × p_2_	Calculated using the formula described in (72, 74)
Effective daily contacts between susceptible pigs and infectious fomites (shared floor, feeders, drinkers, pen walls, equipment, etc.)	c_2_	Poisson (1)	Assumed contact frequency informed by published ASF transmission evidence (75)
Probability of transmission by c_2_	p_2_	0.007–0.05	Range selected based on published estimates of ASFV transmission probability (77)
Virus shedding rate into the environment (per day)	α	0.5–1.5 Env-units per pig	Range selected based on published ASFV shedding data (78)
Environmental contamination half-life (days)	t1/2	3–14	(79, 80)
Virus decay rate from the environment (per day)	δ	0.05–0.23	Calculated from the environmental half-life ( $\frac{\ln2}{t1/2}$ ) using the relationship described in (53)
Reproductive number of ASF within-farm	R0	βD $+\beta_{e}\frac{\alpha }{\delta }D$	Calculated using the formula described in (72, 74)
Removal rate from the infectious state per day	γ	1/D	Calculated using the formula described in (81)
Disease progression rate from exposed (E) to infectious (I)	σ	1/LP	Calculated using the formula described in (82)
Proportion of infectious pigs that died from ASF (case fatality)	ρ	Number of ASF deaths/number of infectious cases	Calculated using the formula described in (83)
Natural mortality rate (per day)	μ	Fixed (0.001)	Fixed value selected based on published mortality data (84)
Within-farm transmission model (Enhanced biosecurity scenario)			
1. Farm segmentation			
Farm segmentation multiplier	m_seg_	0.4–0.6	Multiplier range selected based on published biosecurity evidence (85, 86)
Pig-to-pig transmission rate after farm segmentation (per day)	β´	(c_1_ × m_seg_)p_1_	Calculated using the scenario-specific formula described in (72, 74)
2. Improved hygiene			
Environmental hygiene multiplier	m_hyg_	0.45–0.65	Multiplier range selected based on published biosecurity evidence (47, 87)
Environment-to-pig transmission rate after hygiene	βe´	(c_2_ × m_hyg_)p_2_	Calculated using the scenario-specific formula described in (72, 74)
Virus decay rate multiplier	δ´	1.5–1.75	Multiplier range selected based on published evidence on ASFV environmental persistence and biosecurity (47, 86)
Virus shedding rate multiplier	α´	0.5–0.7	Multiplier range selected based on published evidence on ASFV shedding and biosecurity (47, 86)
Reproductive number of ASF within-farm after enhanced biosecurity	R0´	β´D $+\beta e^{´}\;\frac{\alpha^{\prime }}{\delta^{\prime }}D$	Calculated using the formula described in (72, 74)
Within-farm transmission model (Outbreak response scenario)			
1. Detection & response timing			
Day when the outbreak is detected after the first infectious case (detection delay)	t_detect_	0–7	(14)
Response delay (days)	r_delay_	2–3	(49)
2. Quarantine / movement restriction strength (implemented as multipliers)			
Pig-to-pig quarantine multiplier	m_quar_	0.3–0.5	Multiplier range selected based on published evidence on ASF control and quarantine measures (85, 86)
Pig-to-pig transmission rate after quarantine (per day)	β´´	(c_1_ × m_quar_)p_1_	Calculated using the formula described in (72, 74)
Environment-to-pig quarantine multiplier	m_quar_env_	0.4–0.6	Multiplier range selected based on published evidence on environmental biosecurity and indirect ASFV transmission (47, 87)
Environment-to-pig transmission rate after enhanced hygiene	βe´´	(c_2_ × m_quar_env_)p_2_	Calculated using the formula described in (72, 74)
3. Enhanced cleaning during response			
Virus decay rate multiplier	δ´´	1.3–1.5	Multiplier range selected based on published evidence on ASFV environmental persistence and biosecurity (47, 86)
Virus shedding rate multiplier	α´´	0.7–0.85	Multiplier range selected based on published evidence on ASFV shedding and biosecurity (47, 86)
Reproductive number of ASF within-farm after implementing outbreak response parameters	R0´´	β´´D $+\beta e^{´´}\;\frac{\alpha^{\prime \prime }}{\delta^{\prime \prime }}D$	Calculated using the formula described in (72, 74)
4. Culling strategy parameters			
The number of infectious pigs that triggered the outbreak response	trigger_I	Fixed (1)	(88, 89)
How much of the farm is culled	cull_fraction	Fixed (1)	(88, 89)
Culling rate (per day)	cull_rate	N/2	(88, 89)

Primes (′ / ″) indicate scenario-specific effective parameters; baseline parameter values are unchanged.

#### 2.1.2 Model entities, state variables, and parameters

The model represents individual pigs as independent agents within a closed farm population. The total number of pigs on the farm (𝑁) was sampled uniformly between 500 and 2,500 to reflect variability in farm size under the South Korean production context (16). At model initialization, all pigs were assumed to be susceptible except for a single infectious pig introduced to begin the outbreak. Each pig was explicitly simulated and assigned a discrete epidemiological state, allowing infection and disease progression to emerge from individual-level interactions.

Each pig could occupy one of four mutually exclusive disease states, i.e., SEIR (Figure 1). Susceptible pigs were at risk of infection through either direct contact with infectious pigs or indirect exposure to environmental contamination. Exposed pigs were infected but not yet infectious and progressed to the infectious state following a latent period. Infectious pigs actively transmitted the virus to other pigs and contributed to environmental contamination. Removed pigs were animals that died from ASF or were culled and no longer contributed to transmission. In addition to disease-related transitions, a constant natural mortality rate independent of ASF infection was applied to all pigs (Table 1).

Infection of susceptible pigs was governed by a hazard-based force of infection that combined direct and indirect transmission pathways. This infection pressure, arising from contact with infectious pigs and environmental contamination, was defined as follows (Equation 1):

$$\lambda_{\text{direct}}=\beta \times \frac{I_{t}}{N},\lambda_{\text{indirect}}=\beta_{e}\times \frac{\mathrm{En}v_{t}}{N}\;$$ (1)

where β and β_e_ are the pig-to-pig and environment-to-pig transmission rates, respectively. I_t_ is the number of infectious pigs at time *t*, Env_t_ is the level of environmental contamination, and 𝑁 is the total number of pigs on the farm. The total force of infection acting on each susceptible pig at time *t* was calculated using Equation 2:

$$\lambda_{t}=\beta \frac{I_{t}}{N}+\beta_{e}\frac{\mathrm{En}v_{t}}{N}$$ (2)

The daily probability of infection was derived from this hazard, using an exponential survival function, as shown in Equation 3:

$$P{\left(\text{infection}\right)}_{t}=1-e^{-\mathrm{\lambda t}}\;$$ (3)

This allows infection risk to accumulate continuously over time while being implemented within a discrete daily time step.

Disease progression occurred probabilistically at the agent level. Exposed pigs transitioned to the infectious state with a daily probability of 1–e^–σ^, where σ is the inverse of the latent period. Infectious pigs left the infectious state with a daily probability of 1–e^–γρ^, where γ is the inverse of the duration of infectiousness, and *ρ* represents the proportion of infectious pigs that die from ASF. Given the high case fatality of ASF, removal was assumed to represent ASF-related mortality or culling. Natural mortality was applied to all non-removed pigs at a constant daily probability of 1–e^–*μ*^.

At the farm level, environmental contamination (Env_t_) was represented as a continuous state variable capturing indirect transmission through contaminated fomites, housing surfaces, or shared equipment (Figure 1). Environmental contamination accumulated through viral shedding from infectious pigs and decayed over time as described by Equation 4:

$$\frac{\mathrm{dEn}v_{t}}{\mathrm{dt}}=\alpha I_{t}-\mathrm{\delta En}v_{t}$$ (4)

where *α* is the virus shedding rate into the environment, and *δ* is the virus decay rate, derived from the environmental half-life of ASF virus to ensure consistency with empirical estimates.

All epidemiological and transmission-related parameters were derived from published studies or selected as biologically plausible, literature-informed assumptions relevant to ASF transmission (Table 1). Where direct parameter estimates were unavailable, parameter ranges or scenario multipliers were selected based on information reported in the cited literature rather than through formal statistical calibration or numerical optimization. Transmission rates were expressed as the product of effective contact rates and per-contact transmission probabilities to ensure biological interpretability. Parameter values were sampled from predefined ranges to capture uncertainty, and scenario-specific modifications were implemented through multiplicative adjustments to baseline parameters, as indicated by prime notation in Table 1.

#### 2.1.3 Scenario analysis

To evaluate the potential impact of control measures on ASF dynamics, a set of intervention scenarios was developed by modifying selected parameters of a baseline model to generate counterfactual outcomes (Table 1). The baseline scenario represents ASF transmission under routine farm management conditions, without additional biosecurity enhancements or outbreak response interventions. Transmission occurs through direct pig-to-pig contact and indirect exposure through environmental contamination, with all parameters sampled from the baseline ranges (Supplementary Table S1). Building on this baseline, two intervention scenarios were constructed, namely an enhanced biosecurity scenario and an outbreak response scenario. All scenarios shared the same underlying model structure, with differences implemented through scenario-specific modifications to transmission, environmental, and control-related parameters.

The enhanced biosecurity scenario represents the implementation of preventive control measures prior to outbreak detection. These measures include farm segmentation and improved hygiene practices, modeled as multiplicative reductions in effective contact rates and environmental transmission, alongside increased viral decay and reduced virus shedding into the environment. Scenario-specific parameter adjustments were applied using predefined multipliers, while all remaining parameters followed baseline assumptions (Supplementary Table S2). The outbreak response scenario represents reactive control measures implemented following ASF detection on the farm. This scenario incorporates detection delays, movement restrictions, enhanced cleaning, and culling strategies. Upon detection, transmission parameters were reduced using quarantine-related multipliers, environmental decay rates were increased, and virus shedding rates were reduced. Culling was triggered when the number of infectious pigs exceeded a predefined threshold, with the rate and extent of culling specified in Supplementary Table S3. Parameter values were sampled from predefined ranges to capture uncertainty, and scenario-specific modifications were implemented through multiplicative adjustments to baseline parameters, as indicated by prime notation in Table 1.

#### 2.1.4 Sensitivity analysis

To quantify the influence of model input parameters on key within-farm epidemic outcomes under baseline conditions, a global sensitivity analysis was conducted using partial rank correlation coefficients (PRCC). Parameter sets were generated using Latin hypercube sampling (LHS) across the predefined baseline parameter ranges specified in Table 1, and the baseline model was simulated 1,000 times. PRCCs quantify the strength and direction of monotonic relationships between input parameters and model outputs while controlling for the effects of other parameters. PRCC was selected as an appropriate method given the expected monotonic relationships between epidemiological parameters and model outcomes. The PRCC analysis was applied to four epidemiological outcomes: the basic reproductive number (R0), representing the average number of secondary infections generated by a single infectious pig in a fully susceptible farm population, accounting for both direct pig-to-pig transmission and indirect transmission through environmental contamination; epidemic duration, measured as the number of days the ASF epidemic persisted in the simulated herd; cumulative mortality, expressed as the proportion of pigs that died by the end of the simulation; and peak prevalence, defined as the maximum proportion of pigs infected at any single time point during the epidemic.

Before PRCC estimation, rank transformation was applied to both input parameters and model outputs to reduce sensitivity to non-normality and nonlinear relationships. PRCC values were computed in R using the *sensitivity* package, with 95% bootstrap confidence intervals estimated from 2,000 bootstrap replicates. Two-sided *p*-values were calculated for each PRCC estimate, with statistical significance defined as *p* < 0.05. All analyses were based on 1,000 baseline simulation runs. Results were summarized using PRCC estimates, 95% confidence intervals, *p*-values, and sample size, and were visualized using tornado plots to facilitate comparison of parameter influence across the four epidemic outcomes. Parameters with larger absolute PRCC values were interpreted as having stronger associations with the corresponding outcome when their 95% confidence intervals excluded zero and *p* < 0.05. The sign of statistically supported PRCC estimates was used to indicate the direction of association. For estimates with confidence intervals spanning zero and *p* ≥ 0.05, no directional association was inferred.

### 2.2 Between-farm outbreak analysis

#### 2.2.1 Study area and data sources

To characterize between-farm outbreak patterns of ASF in South Korea, a spatiotemporal dataset of confirmed outbreaks in domestic pig farms from 2019 to 2026 was assembled and analyzed. This dataset integrates outbreak-level information on geographic location, date of confirmation, and farm characteristics, enabling reconstruction of the spatial and temporal progression of ASF at the national scale (Supplementary Table S5). Temporal patterns of ASF occurrence were quantified using aggregated annual and monthly outbreak counts (Supplementary Table S6), providing a descriptive overview of epidemic intensity over time. Given the marked increase in outbreak frequency observed in 2026, this year was selected for detailed outbreak-pattern analysis. A subset of outbreak data corresponding to 2026 was extracted (Supplementary Table S7), and a relative temporal metric (confirmation day) was defined as the number of days since the first confirmed outbreak of the year, enabling consistent temporal ordering of cases. To provide epidemiological context, provincial-level data on pig farm distribution and pig population sizes in 2026 were compiled (Table 2). These data were used to support the interpretation of observed spatial patterns, although complete geolocation data for all farms were not available.

**Table 2:** Provincial distribution of pig farms, pig population, and ASF–infected farms and pigs in South Korea in 2026.

Province	Total number of pig farms	Number of infected pig farms in 2026	Total pig population	Infected pig population
Gyeonggi-do	1,107	7	1,760,281	28,730
Chungcheongnam-do	994	3	2,178,031	11,623
Jeollabuk-do	635	2	1,235,951	22,540
Gyeongsangnam-do	605	5	1,305,183	25,614
Gyeongsangbuk-do	604	1	1,287,349	2,759
Jeollanam-do	490	4	1,143,018	28,427
Chungcheongbuk-do	287	—	600,196	—
Jeju-do	260	—	535,033	—
Gangwon-do	235	2	472,404	24,500
Daegu	52	—	108,650	—
Sejong	48	—	94,863	—
Incheon	26	—	23,298	—
Ulsan	21	—	35,339	—
Busan	12	—	4,179	—
Gwangju	8	—	7,786	—
Daejeon	1	—	0	—

The single registered pig farm in Daejeon was inactive/depopulated during the reporting period; therefore, the number of registered farms was 1, while the recorded pig population was 0 (Ministry of Data and Statistics, Livestock Statistics Survey, 2026).

#### 2.2.2 Description of ASF outbreaks, 2019–2026

To characterize the spatiotemporal dynamics of ASF outbreaks in domestic pig farms, outbreak occurrences from 2019 to 2026 were summarized using both temporal aggregation and spatial visualization approaches. This analysis was conducted to provide a structured overview of outbreak progression and to inform the subsequent distance-based analysis of the 2026 epidemic. Temporal trends were assessed using annual and monthly outbreak counts (Supplementary Table S6), which were used to construct epidemic curves. These curves enabled examination of inter-annual variability in outbreak intensity, as well as intra-annual patterns, including potential temporal clustering and seasonal fluctuations in outbreak occurrence. Spatial patterns were examined using GIS-based mapping of outbreak locations at the administrative unit level. The cumulative distribution map illustrates the geographic extent of ASF outbreaks across the study period, while annual maps depict changes in spatial distribution over time. These visualizations allow identification of regions with repeated outbreak occurrence and provide insight into the spatial expansion of ASF within South Korea. Together, these temporal and spatial summaries provide a descriptive foundation for understanding ASF outbreak dynamics from 2019 to 2026 and establish the basis for the focused analysis of the 2026 epidemic.

#### 2.2.3 The 2026 epidemic: peak outbreaks and spatial expansion

The 2026 epidemic was selected for detailed analysis due to its markedly elevated outbreak intensity and broader spatial distribution compared to previous years. Notably, the available data for 2026 extends only to March, suggesting that the observed outbreak count likely underestimates the full extent of epidemic activity for the year. The spatiotemporal progression of outbreaks during 2026 was visualized using a GIS-based mapping approach, in which each outbreak is represented by its location at the administrative unit level and annotated with its order of occurrence and date of confirmation. This visualization enables simultaneous assessment of the chronological sequence of outbreaks and their spatial distribution, highlighting both localized clustering and occurrences at considerable distances within the same epidemic period.

#### 2.2.4 Distance calculation between confirmed outbreak farms

To describe the spatial relationship among confirmed ASF outbreaks in domestic pig farms during 2026, distances were calculated between temporally ordered outbreak farms. The analysis was based on confirmed ASF outbreaks reported in South Korea in 2026 (Supplementary Table S7), for which geographic coordinates and dates of confirmation were available. Because complete geolocation data for all susceptible and uninfected pig farms were not available, the analysis was not designed to estimate absolute farm-level infection risk or to identify confirmed transmission links. Instead, it was used as a descriptive approach to assess whether newly confirmed outbreaks tended to occur near previously confirmed outbreaks or exhibited broader spatial dispersion. Pairwise distances between confirmed outbreak farms were calculated using the great-circle distance derived from geographic coordinates (Supplementary Table S8). Specifically, the distance *d_ij_* represents the spatial separation between a newly confirmed outbreak farm (*i*) and a previously confirmed outbreak farm (*j*), computed using the Haversine formula and expressed in kilometers.

To preserve temporal ordering, only pairs in which farm *j* was confirmed before farm *i* were considered. For each newly confirmed outbreak, distances to all previously confirmed outbreak farms were calculated, and the distance to the nearest previously confirmed outbreak farm was extracted as a summary measure of local spatial proximity. Because the first confirmed outbreak of 2026 had no previously confirmed outbreak farm within that year for comparison, nearest-previous-outbreak distance could be calculated for 23 of the 24 confirmed outbreaks. The resulting distance distribution was used to characterize the spatial pattern of confirmed outbreaks during the 2026 epidemic. Short nearest-neighbor distances were interpreted as consistent with local clustering of confirmed outbreaks, whereas large nearest-neighbor distances were interpreted as evidence of geographically dispersed outbreak occurrence. However, because the true infection source for each farm was unknown, these distances were not interpreted as confirmed transmission distances.

## 3 Results and discussion

### 3.1 Within-farm transmission dynamics under the baseline scenario

#### 3.1.1 Basic reproductive number: uncertainty and sensitivity analysis

The baseline distribution of *R0* was right-skewed, with a median of 2.0 and a 95% prediction interval of 0.00–13.33 (Figure 2a). In 70.3% of simulations, *R0* exceeded 1, indicating that sustained within-farm transmission was likely under most sampled parameter combinations, although stochastic fade-out remained possible under less favorable conditions. Compared with published ASF estimates, this value is lower than the *R0* reported for fattening pigs in Vietnam (3.8–4.76) (34, 35), smallholder free-range herds in Uganda 3.24 (3.21–3.27) (36), experimental pig-to-pig transmission in Georgia 5.3 (1.7–10.3) (37), and estimates from Russia 7.46 (5.68–9.21) (38), suggesting that the transmission potential in the present Korean model is moderate rather than extreme. However, direct numerical comparison across studies should be interpreted cautiously because *R0* is highly context-dependent and varies with production system, outbreak stage, farm structure, and control practices (39, 40). Sensitivity analysis showed that environment-to-pig transmission (*βe*) was the strongest positive driver of R0 (PRCC = 0.901, 95% CI: 0.890–0.914, *p* < 0.001), followed by pig-to-pig transmission (*β*) (PRCC = 0.770, 95% CI: 0.749–0.791, *p* < 0.001). In contrast, virus decay rate was significantly negatively associated with *R0* (PRCC = −0.427, 95% CI: −0.483 to −0.371, *p* < 0.001) (Figure 3a; Supplementary Table S4). This pattern indicates that prolonged environmental survival of the ASF virus substantially enhances the opportunity for susceptible pigs to encounter infectious material, thereby sustaining transmission even when direct contact rates are moderate, which aligns with experimental evidence of ASFV stability in contaminated environments (41, 42). Consequently, measures that accelerate virus inactivation, such as thorough cleaning and disinfection, effective manure handling, and minimizing wet, organic residues, are likely to reduce within-farm epidemic potential in Korean pig farms (43).

**Figure 2:**
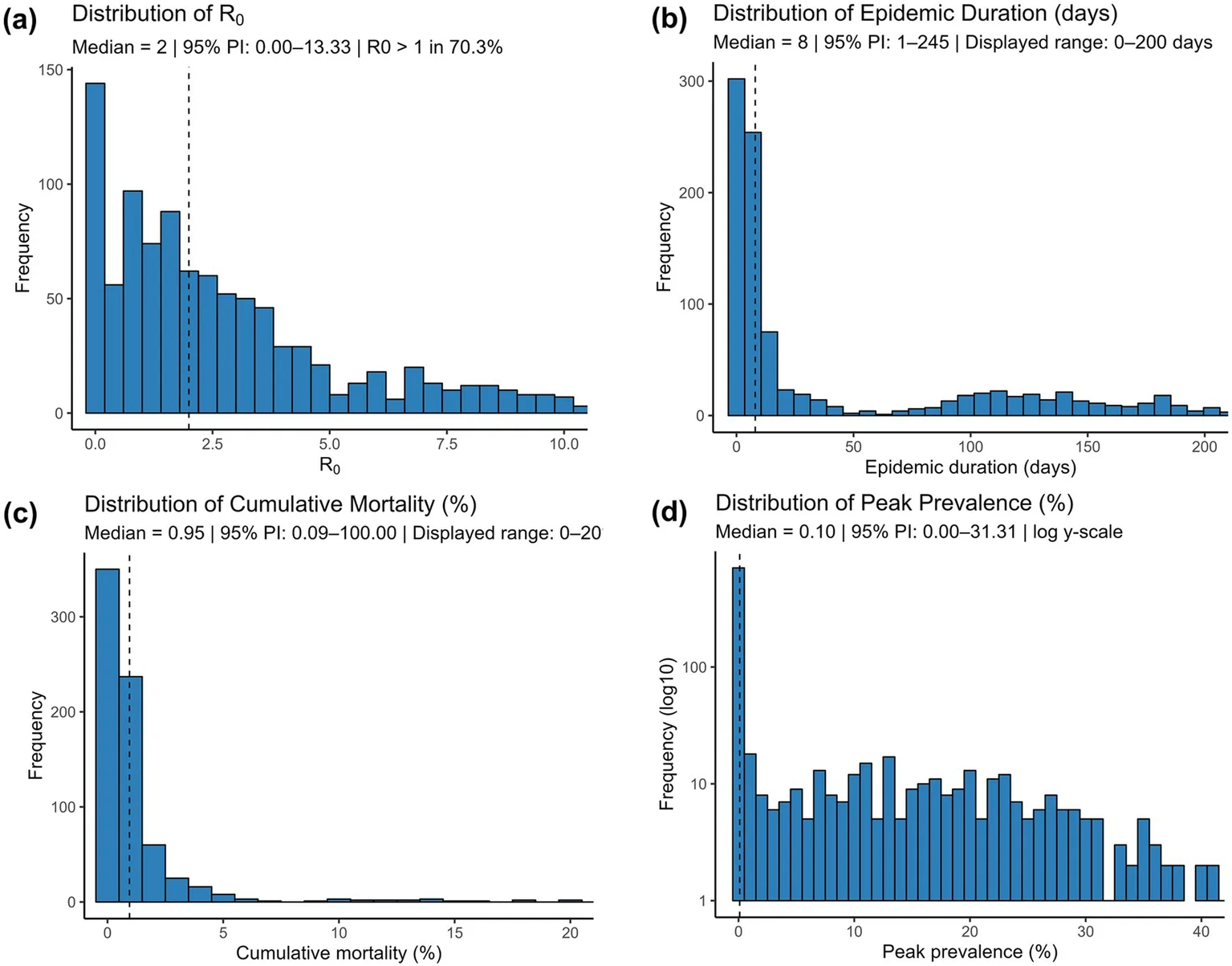
Distributions of within-farm epidemic outcomes from the agent-based model simulations under the baseline scenario. **(a)** Basic reproductive number (R0), **(b)** epidemic duration, **(c)** cumulative mortality, and **(d)** peak prevalence. Dashed vertical lines indicate median values. PI = prediction interval.

**Figure 3:**
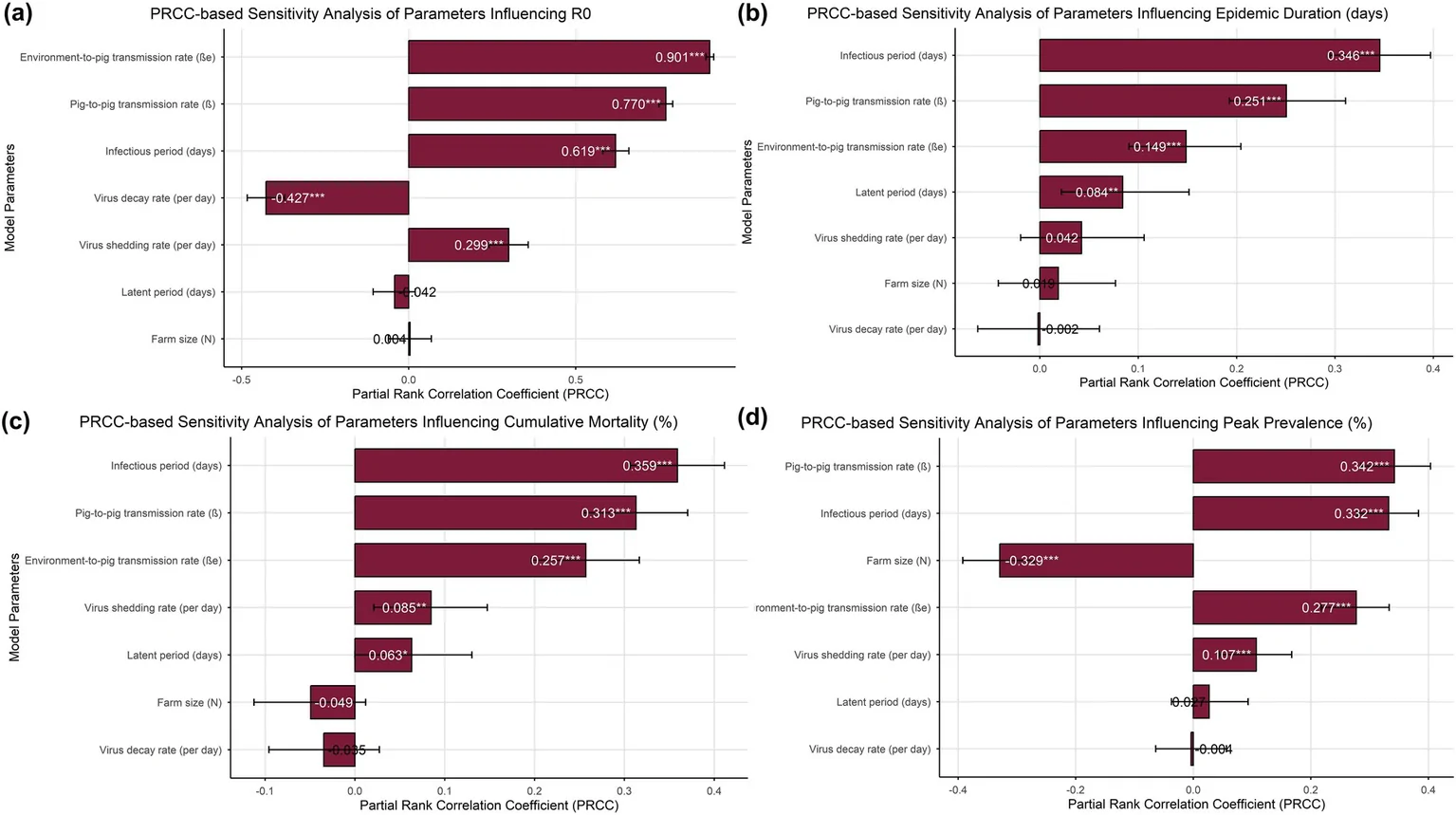
PRCC-based sensitivity analysis of model parameters influencing within-farm epidemic outcomes under the baseline scenario. **(a)** Basic reproductive number (R0), **(b)** epidemic duration, **(c)** cumulative mortality, and **(d)** peak prevalence. Directional interpretations are restricted to PRCC estimates with 95% confidence intervals excluding zero and *p* < 0.05. No directional association is inferred when the confidence interval spans zero and the *p*-value is non-significant.

#### 3.1.2 Epidemic duration: uncertainty and sensitivity analysis

The distribution of epidemic duration under the baseline scenario was right-skewed (Figure 2b). The median duration was 8 days, with a 95% prediction interval of 1–245 days. A considerable proportion of simulations resulted in short epidemic durations, indicating that infection was frequently eliminated within a few days when transmission failed to generate sufficient secondary cases to maintain propagation. This is consistent with observations from the 2019 Korean outbreaks, where rapid detection and stamping out limited farm-level outbreaks to roughly one month or less (11, 21). Conversely, a subset of simulations produced substantially prolonged epidemics, demonstrating that under higher transmission efficiency and continued case generation, infection could persist within the herd for extended periods, as has been suggested by experimental and modeling work on the Georgia 2007/1 strain and field observations in settings with weaker control (25, 37).

Sensitivity analysis revealed that the infectious period was the strongest positive determinant of epidemic duration (PRCC = 0.346, 95% CI: 0.295–0.397, *p* < 0.001), followed by the pig-to-pig transmission rate (PRCC = 0.251, 95% CI: 0.193–0.311, *p* < 0.001) and environment-to-pig transmission (PRCC = 0.149, 95% CI: 0.091–0.204, *p* < 0.001). The latent period also showed a smaller but statistically significant positive association (PRCC = 0.084, 95% CI: 0.022–0.152, *p* = 0.008). In contrast, virus shedding rate (PRCC = 0.042, 95% CI: −0.020–0.106, *p* = 0.182), farm size (PRCC = 0.019, 95% CI: −0.042–0.077, *p* = 0.557), and virus decay rate (PRCC = −0.002, 95% CI: −0.063–0.061, *p* = 0.952) were not significantly associated with epidemic duration (Figure 3b; Supplementary Table S4). These findings indicate that while most outbreaks are short-lived under baseline conditions, epidemic duration remains highly sensitive to how long pigs stay infectious and to the efficiency of direct and indirect transmission. Experimental studies have shown that shortening the infectious period substantially increases the probability of early fade-out and incomplete herd infection, and Korean farm investigations highlight the role of early detection and rapid culling in terminating outbreaks (37). Together, this suggests that interventions that reduce the infectious period (e.g., prompt case recognition, confirmatory testing, and immediate depopulation) and that limit environmental persistence of ASFV through cleaning and disinfection are critical to preventing prolonged within-farm transmission (14, 43).

#### 3.1.3 Cumulative mortality: uncertainty and sensitivity analysis

The distribution of cumulative mortality under the baseline scenario was strongly bimodal (Figure 2c). The median cumulative mortality was 0.95%, with a 95% prediction interval of 0.09–100%. A substantial proportion of simulations resulted in minimal mortality, indicating that infection frequently failed to propagate beyond the initial cases and died out stochastically. In contrast, once transmission chains were successfully established, infection spread extensively through the herd, leading to near-complete mortality. This pattern mirrors the well-recognized “all-or-nothing” behavior of highly virulent ASF viruses, which often cause very high herd-level mortality when uncontrolled but may produce limited losses when rapid depopulation is implemented (25, 44). The limited occurrence of intermediate mortality levels in the model reflects threshold-dependent stochastic dynamics, whereby outbreaks in fully susceptible populations either terminate early or progress to widespread infection.

Sensitivity analysis identified the infectious period as the strongest positive determinant of cumulative mortality (PRCC = 0.359, 95% CI: [0.306–0.411], *p* < 0.001), followed by the pig-to-pig transmission rate (PRCC = 0.313, 95% CI: [0.254–0.371], *p* < 0.001) and environment-to-pig transmission rate (PRCC = 0.257, 95% CI: [0.197–0.317], *p* < 0.001) (Figure 3c; Supplementary Table S4). These parameters directly increase transmission efficiency and the opportunity for secondary case generation, thereby promoting more extensive within-herd spread and higher final mortality, consistent with experimental transmission studies of the Georgia 2007/1 strain and observational data from heavily affected regions (37, 45). In contrast, no detectable association was observed between virus decay rate and cumulative mortality (PRCC = −0.035, 95% CI: −0.096 to 0.027, *p* = 0.275) (Figure 3c; Supplementary Table S4). Although faster environmental inactivation could plausibly reduce indirect transmission based on the known environmental persistence of ASFV (42, 44), this hypothesized relationship with cumulative mortality was not supported by the present sensitivity analysis. Farm size was also negatively associated with proportional mortality, suggesting that in larger herds, infection may not consistently reach all animals before control measures or depletion of susceptible pigs curtail further transmission, a pattern compatible with reports from commercial farms where rapid culling and compartmentalization can prevent complete herd loss despite the high virulence of ASFV (11, 46).

#### 3.1.4 Peak prevalence: uncertainty and sensitivity analysis

The distribution of peak prevalence under the baseline scenario was highly right-skewed and strongly zero-inflated (Figure 2d). The median peak prevalence was 0.10%, with a 95% prediction interval of 0.00–31.31%. The predominance of very low peak values indicates that in a large proportion of simulations, infection failed to spread extensively and resulted in minimal within-farm transmission, consistent with frequent stochastic fade-out after introduction. However, a subset of simulations produced substantially higher peak prevalence, reflecting conditions under which sustained transmission occurred and infection spread to a larger proportion of the herd. This behavior is consistent with observations that highly virulent genotype II/Georgia 2007–like strains can generate a rapid buildup of clinical cases and a heavy within-herd burden when not controlled promptly (25, 37). The wide prediction interval therefore highlights considerable variability in epidemic intensity under plausible combinations of biological parameters and control performance.

Sensitivity analysis demonstrated that the pig-to-pig transmission rate (*β*) was the strongest positive determinant of peak prevalence (PRCC = 0.342, 95% CI: 0.285–0.404, *p* < 0.001), followed by the infectious period (PRCC = 0.332, 95% CI: 0.279–0.383, *p* < 0.001) and the environment-to-pig transmission rate (*βe*) (PRCC = 0.277, 95% CI: 0.218–0.333, *p* < 0.001) (Figure 3d; Supplementary Table S4). These parameters increase transmission efficiency and prolong infectiousness, thereby promoting greater temporal overlap among infectious pigs and elevating the maximum proportion infected at a single time point, in agreement with experimental pig-to-pig transmission studies that report explosive outbreak dynamics under high contact rates and longer infectious periods (37, 45). In contrast, no detectable association was observed between virus decay rate and peak prevalence (PRCC = −0.004, 95% CI: −0.064 to 0.057, *p* = 0.905) (Figure 3d; Supplementary Table S4). Although faster environmental inactivation could plausibly reduce sustained indirect exposure based on the known environmental stability of ASFV (42, 44), this hypothesized relationship with peak prevalence was not supported by the present sensitivity analysis. Collectively, these findings suggest that peak prevalence is primarily governed by transmission efficiency and the duration of infectiousness within the herd, and that interventions which simultaneously reduce effective contacts, shorten the infectious period through rapid detection and culling, and enhance cleaning and disinfection are critical to preventing large within-farm peaks of ASF (14, 43).

### 3.2 Within-farm transmission dynamics under the enhanced biosecurity scenario

Implementation of the enhanced biosecurity scenario resulted in marked reductions in transmission intensity and epidemic severity compared with the baseline scenario (Figure 4). The distribution of *R0* shifted downward, with the median falling below 1, indicating that in most simulations the infection was unable to sustain transmission within the farm. Occasional simulations still produced *R0* > 1, but the overall transmission potential was substantially reduced relative to baseline (Figure 4a). This pattern is consistent with observational evidence that improvements in hygiene, restriction of farm access, and better management of contaminated materials can lower the effective reproduction number of ASFV and prevent self-sustaining outbreaks in commercial pig systems (47). Similar reductions in transmission following enhanced biosecurity have been reported in mechanistic analyses of ASF control in Korea and in other intensive production settings, emphasizing the central role of structural biosecurity in outbreak prevention (48).

**Figure 4:**
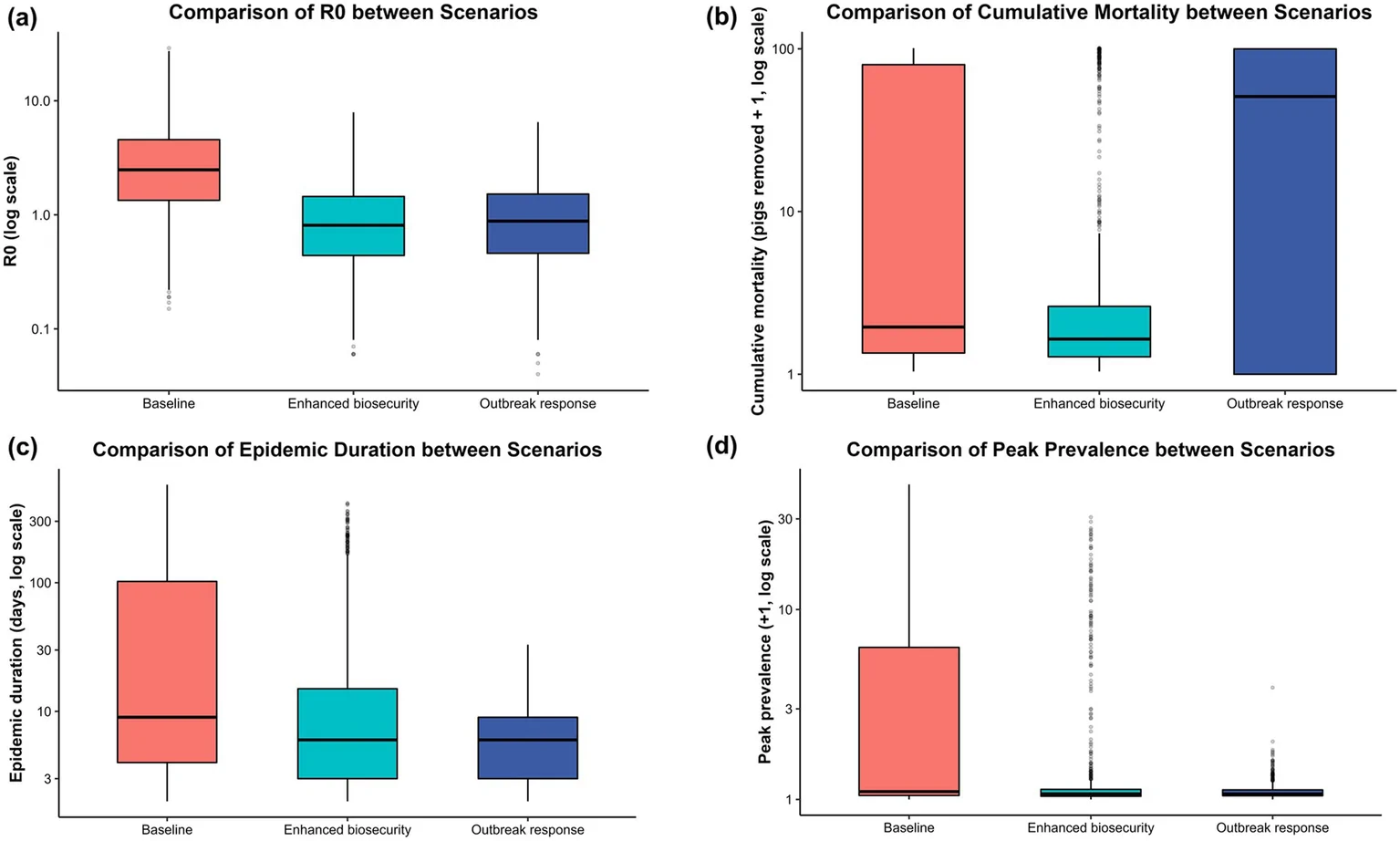
Comparison of within-farm epidemic outcomes under the baseline, enhanced biosecurity, and outbreak response scenarios. **(a)** Basic reproductive number (R0), **(b)** cumulative mortality, **(c)** epidemic duration, and **(d)** peak prevalence. Boxplots show the distribution of simulation outcomes across parameter sets; values are displayed on a log scale where indicated.

Cumulative mortality was also markedly lower under enhanced biosecurity. Although the distribution remained right-skewed, the majority of simulations resulted in very low mortality, typically involving only a small number of pigs, and large-scale mortality events were rare compared with baseline conditions (Figure 4b). This aligns with experiences from countries where strict farm-level biosecurity and rapid on-farm response have limited realized losses despite circulation of highly virulent genotype II viruses (21). Epidemic duration was shortened under enhanced biosecurity, with most outbreaks resolving within a short period and a compressed interquartile range compared with baseline (Figure 4c). These shorter, smaller outbreaks are consistent with farm investigations in Korea and Europe showing that improved surveillance, restricted entry, and routine cleaning can curtail within-farm ASF transmission once infection is introduced (11).

Similarly, peak prevalence remained low across most simulations, with the distribution clustering near minimal values and only sporadic simulations exhibiting moderate increases (Figure 4d). This suggests that enhanced biosecurity not only reduces the probability that ASF will establish but also limits the maximum instantaneous burden of infectious pigs during outbreaks, which is critical for minimizing virus shedding into the environment and reducing the risk of onward spread between farms (44). Overall, the enhanced biosecurity scenario effectively reduced transmission intensity, outbreak size (total pigs removed), and epidemic persistence within farms, reinforcing empirical recommendations that structural biosecurity, such as controlled entry, dedicated clothing and equipment, cleaning and disinfection protocols, and manure management, forms the cornerstone of ASF prevention in domestic pig herds (42).

### 3.3 Within-farm transmission dynamics under the outbreak response scenario

Under the outbreak response scenario, transmission dynamics differed from the enhanced biosecurity scenario but still demonstrated improvement relative to baseline (Figure 4). The median (R0) remained close to 1 but was lower than under baseline conditions, suggesting that while transmission could still occur, epidemic growth was more limited (Figure 4a). Cumulative mortality displayed a bimodal-like pattern. In many simulations, early intervention led to minimal mortality; however, when transmission occurred before control measures took effect, substantial mortality was observed. This resulted in a wider variability in outbreak size compared to the enhanced biosecurity scenario (Figure 4b). Epidemic duration was generally shorter than baseline but comparable to or slightly longer than enhanced biosecurity in some simulations. Most outbreaks were contained within a limited timeframe, reflecting the impact of reactive control measures, though occasional extended outbreaks were observed when early spread was not immediately curtailed (Figure 4c) (14, 49). Peak prevalence was typically low, clustering near minimal levels, but showed less extreme right-tail behavior compared with baseline. This suggests that outbreak response measures were effective in limiting the maximum proportion of infectious pigs at any given time, even in scenarios where transmission initially occurred (Figure 4d) (49). Overall, the outbreak response scenario reduced outbreak magnitude and duration relative to baseline conditions, though variability remained higher than under enhanced biosecurity, reflecting the reactive nature of control measures (50).

### 3.4 Between-farm outbreak patterns

#### 3.4.1 Spatial distribution of ASF outbreaks

The spatial distribution of ASF outbreaks in domestic pig farms in South Korea from 2019 to 2026 showed a clear clustering pattern, with the greatest burden concentrated in the northwestern part of the country, particularly in Gyeonggi-do and Incheon, and in the northern inland areas of Gangwon-do (Figure 5a). In the early period of the epidemic, cases were largely restricted to the border-adjacent municipalities, including Paju-si, Yeoncheon-gun, Gimpo-si, and Ganghwa-gun, with recurrent involvement of Cheorwon-gun and Hwacheon-gun, indicating a persistent northwestern focus (51, 54). This pattern suggests that confirmed ASF outbreaks were not randomly distributed, but were concentrated in areas with repeated outbreak occurrence and proximity to the northern border region. This early clustering is consistent with the geographic and epidemiological characteristics of the region. The northern outbreak belt lies adjacent to the Korean Demilitarized Zone (DMZ), where wild boar populations may contribute to cross-border viral introduction into South Korea (11, 55). In addition, this region overlaps with the densely populated Seoul Metropolitan Area, which contains a high concentration of pig farms and intensive movement networks. Under such conditions, indirect exposure associated with vehicle movement, farm-to-farm contact, personnel movement, contaminated fomites, or slaughterhouse-linked activities may contribute to outbreak occurrence, particularly where farm density is high and biosecurity breaches can facilitate wider dissemination (22).

**Figure 5:**
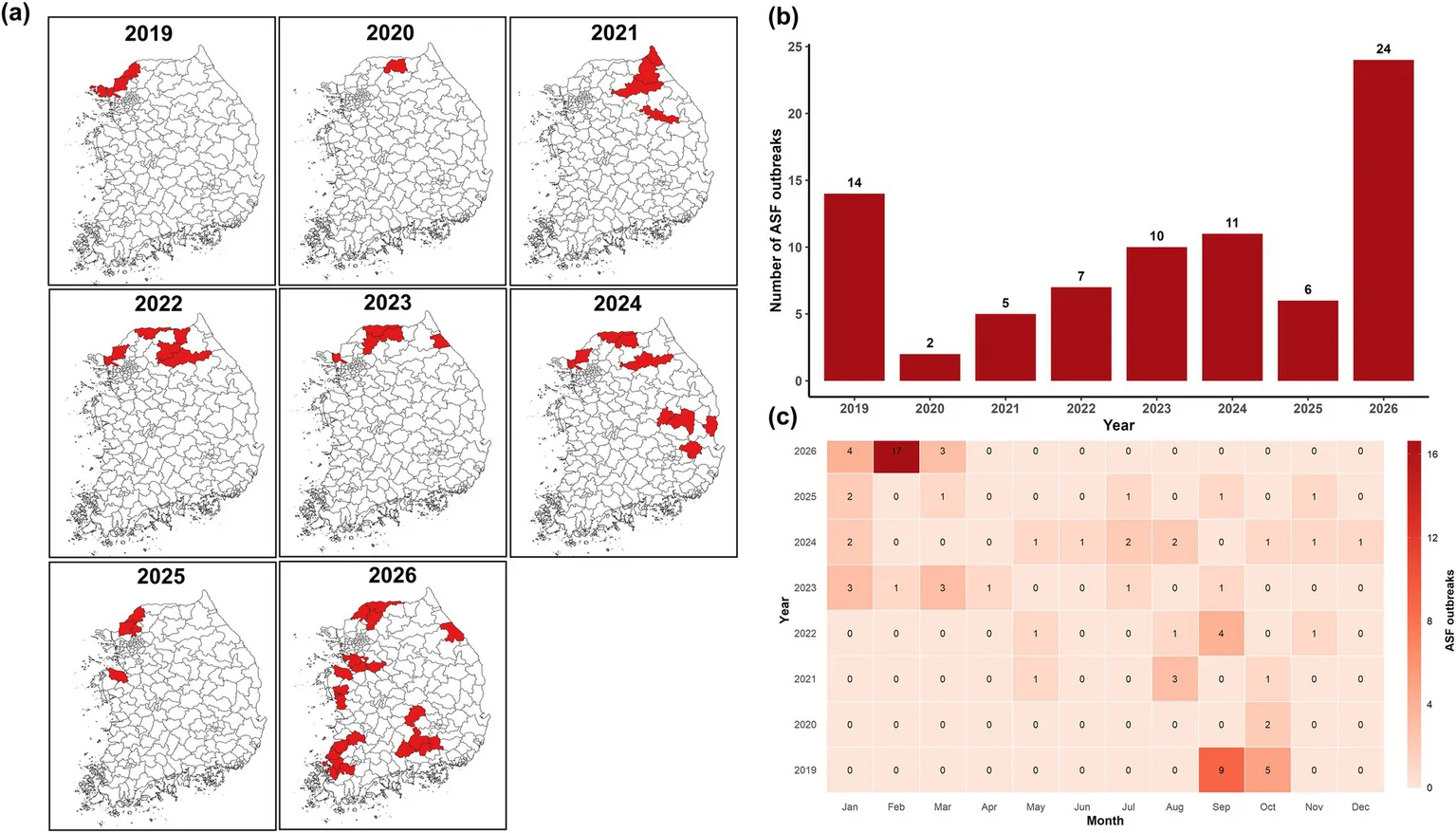
Spatiotemporal distribution of African swine fever (ASF) outbreaks in domestic pig farms in South Korea from 2019 to 2026. **(a)** Annual spatial distribution of ASF outbreaks; red areas indicate administrative units at the city/county level where at least one ASF outbreak was confirmed. **(b)** Annual epidemic curve showing the number of confirmed ASF farm outbreaks by year. **(c)** Year-month heatmap showing monthly counts of confirmed ASF farm outbreaks. Data for 2026 include outbreaks reported up to March.

While the initial phase was characterized by localized clustering, later years showed geographic expansion (Figure 5a). By 2024, outbreaks were no longer confined to the northern frontier and had appeared in more dispersed regions, including Gyeongsangbuk-do and subsequently in southern provinces such as Chungcheongnam-do, Jeollanam-do, Jeollabuk-do, and Gyeongsangnam-do. This broader distribution indicates that confirmed ASF outbreaks were no longer limited to the northern border-associated areas, but were increasingly observed across wider parts of the national swine production system (15). The emergence of outbreaks in previously unaffected areas in 2026 suggests a more geographically dispersed outbreak pattern, potentially involving a combination of local clustering, wildlife-associated risk, delayed detection, and possible non-local production- or movement-associated pathways. Such expansion is compatible with possible non-local connectivity among geographically distant farms, including shared logistics networks, transport vehicles, and contaminated materials such as feed or animal-derived products, although these pathways could not be directly confirmed in the present analysis (22). Notably, recent epidemiological investigations in South Korea have reported the detection of ASF genetic material in porcine plasma-based feed ingredients, raising concerns about potential transmission through the feed supply chain and slaughterhouse-linked contamination pathways, although the presence of infectious virus and its direct role in transmission remain under investigation (56, 57). The 2026 pattern, therefore, indicates increasing epidemiological complexity, with confirmed outbreaks showing both localized clustering and broader geographic dispersion (52).

#### 3.4.2 Temporal patterns of ASF outbreaks

ASF outbreaks in domestic pig farms in South Korea showed substantial temporal variation between 2019 and 2026 at both annual and monthly scales (Supplementary Tables S5, S6). Annual outbreak numbers remained relatively low in 2020–2022 (2–7 outbreaks/year), increased in 2023–2024 (10–11 outbreaks/year), declined in 2025 (6 outbreaks), and showed a pronounced surge in 2026 (24 outbreaks reported by March), representing the highest outbreak count within the study period (Figure 5b). Because 2026 data cover only the first quarter, the final annual total is likely to be even higher, indicating a clear intensification of farm-level activity compared with previous years. This pattern may reflect variation in transmission intensity, the wild boar–domestic pig interface, and the effectiveness of control measures over time (21, 51).

At the monthly scale, ASF outbreaks exhibited a non-uniform temporal distribution, with a clear concentration of cases during the winter and early spring months (Figure 5c). Recurrent outbreak activity was observed in January across multiple years (2023–2026), while additional clustering was evident in March (2023, 2025, and 2026). This pattern was particularly pronounced in 2026, with a substantial increase in outbreaks in January and February, followed by continued activity in March. This seasonal concentration is consistent with findings from other ASF-affected regions, where increased virus persistence and indirect transmission have been associated with low-temperature conditions, including contamination via fomites, carcasses, and movement-related pathways (52, 53). Recent analyses of ASF in Asia, including Korea, have similarly reported higher outbreak frequencies in winter and early spring, particularly in February–March (21, 51). In contrast, outbreaks occurred during the summer and autumn months (e.g., May and August in 2021–2024), and were sporadic and less consistent across years (Figure 5c), indicating reduced seasonal clustering during warmer periods. Overall, these findings support intensified surveillance and biosecurity during January–March, when outbreak risk appears highest (15).

#### 3.4.3 Spatiotemporal progression of the 2026 epidemic

The spatiotemporal progression of confirmed ASF outbreaks in domestic pig farms during 2026 revealed a complex pattern characterized by early multifocal occurrence, subsequent regional clustering, and broader geographic distribution (Supplementary Table S7). The first confirmed outbreak was reported in Gangneung-si (Gangwon-do) on January 17, followed by geographically separated confirmed outbreaks in Anseong-si (Gyeonggi-do) on January 23 and Yeonggwang-gun (Jeollanam-do) on January 26 (Figure 6). This early phase, spanning late January to early February, included confirmed outbreaks across geographically separated regions, including Pocheon-si and Yeoncheon-gun (Gyeonggi-do) in the north and Gochang-gun (Jeollabuk-do) in the southwest. This pattern is compatible with multiple possible explanations, including multiple introductions, delayed detection, unobserved intermediate outbreaks, or non-local dissemination, rather than a clearly observed contiguous local spread pattern (22, 58).

**Figure 6:**
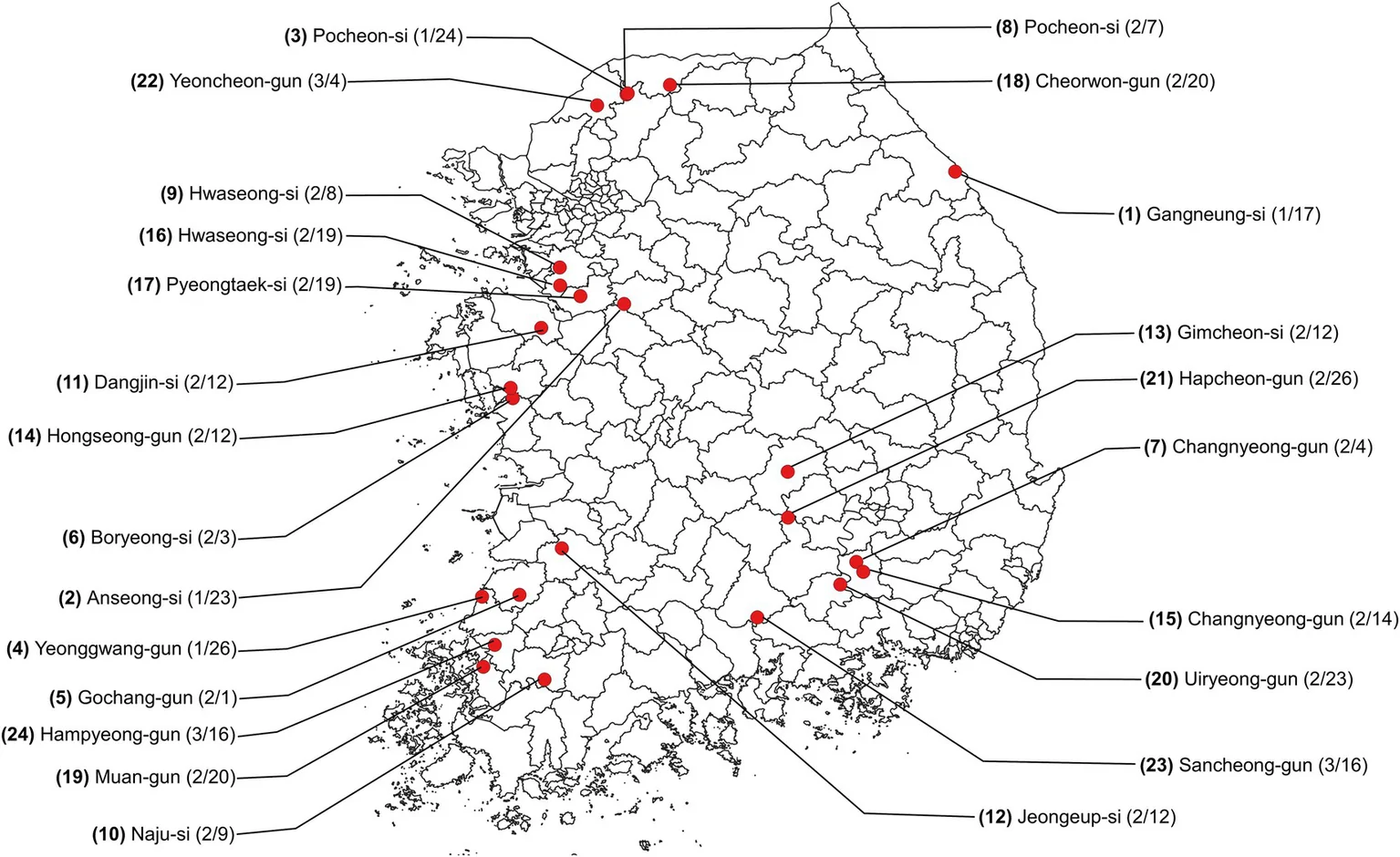
Spatial distribution and temporal progression of confirmed ASF outbreaks in domestic pig farms in South Korea in 2026. Red points indicate the locations of reported outbreaks at the administrative unit level (city/county). Numbers (1–24) represent the chronological order of outbreak confirmation, and the accompanying labels indicate the corresponding administrative unit names and confirmation dates (month/day).

As the epidemic advanced into February, localized clustering became more apparent in the northwest, particularly in Gyeonggi-do and adjacent Chungcheongnam-do, with recurrent confirmed outbreaks in Pocheon-si, Hwaseong-si, Pyeongtaek-si, and Dangjin-si. Concurrently, dispersed outbreaks continued in distant regions, including Boryeong-si and Hongseong-gun (Chungcheongnam-do), Naju-si and Muan-gun (Jeollanam-do), Changnyeong-gun, Uiryeong-gun, and Hapcheon-gun (Gyeongsangnam-do), and Gimcheon-si (Gyeongsangbuk-do). This pattern suggests that the 2026 epidemic involved both localized clustering of confirmed outbreaks and continued occurrence of geographically dispersed outbreaks. Such a pattern is compatible with several possible mechanisms, including local spread, delayed detection, unobserved intermediate outbreaks, and possible non-local production- or movement-associated pathways; however, these mechanisms could not be distinguished using the available data (52).

By late February and early March, this pattern persisted, with northern foci such as Yeoncheon-gun and Cheorwon-gun (Gangwon-do) remaining active alongside newly confirmed outbreaks in southern regions, including Sancheong-gun (Gyeongsangnam-do) and Hampyeong-gun (Jeollanam-do). Overall, the 2026 epidemic showed a transition from early multifocal occurrence to a combined pattern of regional clustering and broader geographic dispersion. This suggests that the spatial arrangement of confirmed outbreaks was not fully described by geographic proximity alone, although the specific mechanisms underlying this pattern could not be confirmed from the available data (22, 58).

#### 3.4.4 Distance-based patterns among temporally ordered confirmed outbreaks

To describe the spatial proximity among confirmed ASF farm outbreaks during the 2026 epidemic, the distance between each newly confirmed outbreak farm and its nearest previously confirmed outbreak farm was calculated (Supplementary Table S9). Given the use of confirmation dates and confirmed outbreak locations, the calculated distances were interpreted as descriptive measures of spatial proximity among temporally ordered confirmed outbreaks, rather than as direct evidence of confirmed farm-to-farm transmission. The distribution of nearest-neighbor distances was highly heterogeneous, with a first quartile of 15.0 km, a median of 31.3 km, and a third quartile of 58.3 km; the mean distance was 52.1 km, and the range extended from <1 km to approximately 190 km (Table 3; Supplementary Table S9). These results indicate that although some confirmed outbreaks occurred close to previously confirmed outbreak farms, a considerable fraction occurred at distances beyond the local ranges typically used for control zoning and spatial models, such as approximately 3–10 km (45, 59).

**Table 3:** Summary statistics of nearest-neighbor distances to the nearest previously confirmed outbreak farm for ASF outbreaks in domestic pig farms in South Korea in 2026.

Statistic	Distance km
Minimum	0.8
1st Quartile (25% of the infections)	15
Median (50% of the infections)	31.3
Mean	52.1
3rd Quartile (75% of the infections)	58.3
Maximum	190

To facilitate interpretation, nearest-neighbor distances were grouped into four descriptive distance categories: <10 km, 10–50 km, 50–100 km, and >100 km (Table 4). Only a small number of outbreaks occurred within <10 km (n = 3), indicating limited short-distance clustering among confirmed outbreaks. In contrast, the majority of outbreaks occurred at distances of 10–50 km (n = 13), showing that many newly confirmed outbreaks were separated from earlier confirmed outbreaks by regional-scale distances. Additional outbreaks were observed at intermediate distances of 50–100 km (n = 3), while a subset of outbreaks occurred at distances exceeding 100 km (n = 4). These findings indicate that the confirmed outbreak pattern was not restricted to close spatial proximity around earlier confirmed farms (60, 61).

**Table 4:** Distribution of confirmed ASF outbreaks according to distance from the nearest previously confirmed outbreak farm in South Korea in 2026.

Distance category	Number of outbreaks
<10 km	3
10–50 km	13
50–100 km	3
>100 km	4

The first confirmed outbreak of 2026 was excluded because no previously confirmed outbreak farm was available for distance calculation; therefore, the distance categories include 23 of the 24 confirmed outbreaks.

Geographically distant confirmed outbreaks were observed from the early phase of the 2026 epidemic. The second, third, and fourth reported outbreaks occurred approximately 178 km, 126 km, and 190 km from their nearest previously confirmed outbreak farms, respectively (Supplementary Table S9). This early appearance of widely separated confirmed outbreaks is compatible with several possible explanations, including multiple introductions, delayed detection, unobserved intermediate outbreaks, or non-local dissemination pathways (52). However, the available data do not allow these alternatives to be distinguished. As the epidemic progressed, shorter nearest-neighbor distances became more frequent, with several outbreaks occurring within 5–10 km of previously confirmed outbreak farms. This pattern suggests that local clustering became more apparent after the initial geographically dispersed outbreak pattern. Overall, these results indicate that confirmed ASF farm outbreaks during the 2026 epidemic showed both local clustering and broader geographic dispersion (52, 62). However, because confirmed transmission links were not available, these findings should be interpreted as descriptive evidence of outbreak spacing rather than direct evidence of specific farm-to-farm transmission pathways.

#### 3.4.5 Potential explanations for geographically dispersed confirmed outbreaks

The spatial and distance-based analyses showed that confirmed outbreaks during the 2026 epidemic occurred across a wide range of distances from previously confirmed outbreak farms. Although some outbreaks occurred within 10 km of a previously confirmed outbreak farm, several were located at substantially larger nearest-neighbor distances, including multiple outbreaks more than 100 km from the nearest previously confirmed outbreak. Because these distances represent spatial proximity among temporally ordered confirmed outbreaks rather than confirmed transmission links, the mechanisms underlying the geographically dispersed outbreak pattern cannot be determined from the available data.

One plausible, but unconfirmed, explanation is the contribution of human-mediated or production-network-associated pathways. In modern pig production systems, farms may be indirectly connected through movements of animals, feed, vehicles, equipment, and personnel (22, 63). Such connections could allow contaminated material to move between geographically distant farms and generate outbreak patterns that deviate from simple spatial proximity. However, because movement records, trace-back information, genomic data, and complete susceptible-farm locations were not available, the present study could not directly test or quantify the contribution of these pathways.

Additional pathways may also be relevant. In particular, the feed supply chain has emerged as a potential concern in South Korea, given recent epidemiological attention to porcine plasma-derived feed ingredients as a possible source of ASF dissemination (56, 57). Importantly, these investigations have reported the molecular detection of ASFV DNA by PCR, which does not confirm the presence of viable or infectious virus capable of causing transmission. Therefore, the epidemiological significance of ASFV DNA detected in feed ingredients remains uncertain, and feed-associated transmission should not be considered a confirmed explanation for the observed outbreak pattern. Nevertheless, the centralized production, transport, and distribution of feed ingredients could provide a biologically plausible pathway for the wider dissemination of contaminated materials. Accordingly, stringent biosecurity measures during feed production, transportation, storage, and distribution remain essential precautions, while the potential role of feed-associated transmission warrants further investigation.

Slaughterhouses may represent another potential interface for geographically dispersed outbreak occurrence. As convergence points linking multiple farms through animal transport and processing activities, these facilities may facilitate indirect transmission if biosecurity is insufficient (64, 65). Contaminated vehicles, equipment, or movement routes associated with slaughterhouse operations could potentially connect farms that are otherwise widely separated in space. However, the present analysis did not directly evaluate slaughterhouse-linked movements, and therefore this pathway cannot be confirmed from the available data.

Another important explanation is that some apparent long-distance separations may reflect delayed detection or unobserved intermediate infections. Under this scenario, the observed pattern may not represent single-step long-distance spread, but rather a sequence of undetected transmission events or differences between actual infection dates and confirmation dates (14, 25). This limitation is particularly important because the analysis was based on confirmation dates rather than estimated infection dates. Therefore, geographically distant confirmed outbreaks should be interpreted cautiously and not assumed to represent direct long-distance transmission events.

Collectively, these findings are compatible with a multi-pathway interpretation of ASF spread, in which local clustering, delayed detection, unobserved intermediate infections, and non-local production-network-associated pathways may all contribute to the observed outbreak pattern. However, the relative contribution of each pathway could not be quantified using the available data. From a control perspective, the results suggest that local containment around confirmed outbreak farms remains essential, but may need to be complemented by broader surveillance and biosecurity measures targeting transport systems, shared service networks, feed distribution pathways, slaughterhouse-associated movements, and other high-connectivity points within the pig production system (60, 63).

## 4 Implications for ASF control in South Korea

Collectively, the within-farm and between-farm components of this study provide complementary guidance for ASF control in South Korea. The within-farm model showed that the infectious period, environmental transmission, and biosecurity strongly influenced outbreak size and duration, underscoring the value of rapid detection, prompt removal of infectious pigs, and rigorous cleaning and disinfection on affected farms. At the same time, the between-farm analyses showed confirmed outbreak patterns characterized by both local clustering and broader geographic dispersion. The occurrence of both locally clustered and geographically dispersed confirmed outbreaks highlights the importance of considering surveillance and biosecurity measures beyond immediate outbreak zones, while recognizing that the underlying transmission pathways could not be determined from the available data.

The occurrence of geographically distant confirmed outbreaks, including several early in the 2026 epidemic, highlights the importance of control strategies that extend beyond immediate outbreak areas. Short-range measures such as rapid culling, quarantine, and movement restrictions within defined buffer zones remain essential for limiting local spread. However, these measures should be accompanied by broader surveillance and biosecurity activities targeting possible non-local routes of outbreak dissemination (22, 52). In this context, strengthening biosecurity across transport and service networks is particularly important. Movements of animals, vehicles, equipment, and personnel between farms represent plausible routes for indirect spread that may connect farms across provinces (37, 63). Enhanced cleaning and disinfection protocols, movement tracking, and regulation of shared service providers may therefore help reduce the risk of geographically dispersed outbreak occurrence.

The findings also support increased attention to high-connectivity points within the production system, such as feed distribution networks and slaughterhouses. Although the present study did not directly evaluate these pathways, previous studies of feed, carcass, and movement systems suggest that such points may contribute to disease dissemination if biosecurity is insufficient (65, 66). In addition, the possibility of delayed detection or unobserved intermediate infections indicates that surveillance systems should prioritize early detection across a wider geographic area rather than focusing exclusively on zones surrounding known outbreaks (14, 25). Coordinated control strategies that combine rapid local containment and on-farm biosecurity with broader risk management at high-connectivity points are therefore likely to be more effective for ASF control in complex pig production systems (60, 63).

## 5 Study limitations and future directions

Although this study provides important insights into simulated within-farm ASF dynamics and observed between-farm outbreak patterns in South Korean pig farms, several limitations should be acknowledged. First, the within-farm agent-based model was parameterized using published ranges and plausible assumptions rather than detailed Korean farm-level outbreak data. Although uncertainty and sensitivity analyses were performed, some processes, particularly environmental contamination and indirect exposure, could not be directly calibrated or validated. In addition, the model represents a simplified commercial herd and does not explicitly capture farm-specific housing structures, pen-level contact patterns, production stages, or biosecurity practices. Second, the between-farm distance-based analysis was based on confirmed outbreak locations and confirmation dates rather than confirmed transmission links or estimated infection dates. Therefore, nearest-neighbor distances and temporally ordered outbreak-pair distances should be interpreted as descriptive measures of spatial proximity among confirmed outbreaks, not as confirmed transmission distances. Because complete geolocation data for susceptible and uninfected farms were unavailable, formal assessment of distance-dependent farm-to-farm transmission was not possible. Without the underlying spatial distribution of farms at risk, the frequency of observed outbreaks across distance ranges cannot be interpreted as distance-specific transmission risk. In addition, detailed movement, trace-back, genomic, feed-distribution, slaughterhouse, and service-network data were unavailable; therefore, the potential contributions of human-mediated or production-network-associated pathways could not be directly tested or quantified. Some geographically distant confirmed outbreaks may also reflect delayed detection or unobserved intermediate outbreaks. Finally, the 2026 data covered only a partial year and a limited number of confirmed outbreaks, which may constrain the generalizability of the distance-based findings. Future studies integrating complete susceptible-farm locations with outbreak data, animal and vehicle movement records, trace-back investigations, service-network information, and genomic or phylodynamic evidence could support formal estimation and validation of distance-dependent transmission kernels and more direct evaluation of ASF transmission pathways.

## 6 Conclusion

This study shows that understanding ASF dynamics in South Korean pig farms requires consideration of both within-farm transmission processes and between-farm outbreak patterns. At the within-farm level, agent-based simulations indicated that outbreak size and duration were strongly influenced by the infectious period, environmental transmission, and biosecurity, highlighting the importance of rapid detection, prompt removal of infectious pigs, and effective cleaning and disinfection. At the between-farm level, confirmed outbreaks in 2026 showed both local clustering and broader geographic dispersion, with several outbreaks located at intermediate or long nearest-neighbor distances from earlier confirmed outbreaks. Together, these findings are compatible with a multi-pathway interpretation of ASF outbreak dynamics, while recognizing that specific non-local mechanisms could not be directly confirmed with the available data. Effective control should therefore combine strong on-farm biosecurity and rapid local containment with broader surveillance and risk management at high-connectivity points within the pig production system.

## Data Availability

The original contributions presented in the study are included in the article/Supplementary material, further inquiries can be directed to the corresponding author.
